# Myoelectric prosthesis users and non-disabled individuals wearing a simulated prosthesis exhibit similar compensatory movement strategies

**DOI:** 10.1186/s12984-021-00855-x

**Published:** 2021-05-01

**Authors:** Heather E. Williams, Craig S. Chapman, Patrick M. Pilarski, Albert H. Vette, Jacqueline S. Hebert

**Affiliations:** 1 Department of Biomedical Engineering, Faculty of Medicine and Dentistry, University of Alberta, Edmonton, AB Canada; 2 Faculty of Kinesiology, Sport, and Recreation, University of Alberta, Edmonton, AB Canada; 3 Department of Medicine, Faculty of Medicine and Dentistry, University of Alberta, Edmonton, AB Canada; 4 Department of Mechanical Engineering, Faculty of Engineering, University of Alberta, Edmonton, AB Canada; 5 Glenrose Rehabilitation Hospital, Alberta Health Services, Edmonton, AB Canada

**Keywords:** Transradial amputation, Compensatory movements, Motion capture, Myoelectric prosthesis, Simulated prosthesis, Bypass prosthesis, Upper body kinematics

## Abstract

**Background:**

Research studies on upper limb prosthesis function often rely on the use of simulated myoelectric prostheses (attached to and operated by individuals with intact limbs), primarily to increase participant sample size. However, it is not known if these devices elicit the same movement strategies as myoelectric prostheses (operated by individuals with amputation). The objective of this study was to address the question of whether non-disabled individuals using simulated prostheses employ the same compensatory movements (measured by hand and upper body kinematics) as individuals who use actual myoelectric prostheses.

**Methods:**

The upper limb movements of two participant groups were investigated: (1) twelve non-disabled individuals wearing a simulated prosthesis, and (2) three individuals with transradial amputation using their custom-fitted myoelectric devices. Motion capture was used for data collection while participants performed a standardized functional task. Performance metrics, hand movements, and upper body angular kinematics were calculated. For each participant group, these measures were compared to those from a normative baseline dataset. Each deviation from normative movement behaviour, by either participant group, indicated that compensatory movements were used during task performance.

**Results:**

Results show that participants using either a simulated or actual myoelectric prosthesis exhibited similar deviations from normative behaviour in phase durations, hand velocities, hand trajectories, number of movement units, grip aperture plateaus, and trunk and shoulder ranges of motion.

**Conclusions:**

This study suggests that the use of a simulated prosthetic device in upper limb research offers a reasonable approximation of compensatory movements employed by a low- to moderately-skilled transradial myoelectric prosthesis user.

## Background

Myoelectric prostheses are used to restore or improve impaired arm and hand function so that individuals with upper limb amputation can independently accomplish activities of daily living [1]. Device use requires individuals to adapt their movement strategies, particularly when executing tasks that involve object manipulation [2–4]. Such adaptations can include the introduction of additional trunk and shoulder movements to better control the positioning of the prosthesis [3, 4]. These additional movements that are not normally observed in those with typical arm function are referred to as “compensatory movements”. These compensations make it more laborious to use the prosthesis and are one cited reason for device rejection [3, 5]. Innovations in prosthetic design and control have attempted to improve device usability and reduce compensations. However, the ability to statistically measure the effects of such advances is limited by the relatively small [6] and heterogenous population of individuals with major upper limb amputation [6, 7].

To assess new myoelectric prosthesis control methods, researchers often use a simulated prosthetic device worn by non-disabled participants [8–11]. A simulated device allows these participants to control a myoelectric prosthetic hand in the same manner as an individual with a transradial amputation, that is, through activation of their forearm muscles. Simulated prostheses generally consist of a brace that attaches to a research participant’s forearm, with a prosthetic terminal device (hand or hook) extending distally or offset to the dorsal, palmar, or radial side of their hand [8, 12]. The benefits of using a simulated device in research are two-fold. Firstly, it allows for the recruitment of a larger number of participants, which improves the statistical power of the research findings [8]. Secondly, the extent of device training experience that participants using a simulated prosthesis receive prior to data collection can be controlled. Typically, individuals with transradial amputation exhibit a wide range of device use experience [13–17].

Simulated prostheses have been used to study control system alternatives [18–21], hand–eye coordination [22], sensory feedback systems [23–25], and compensatory movements [26–28]. The majority of these studies have used validated functional task assessments such as the Southampton Hand Assessment Procedure (SHAP) [9–11, 19, 20, 29–32], the Box and Blocks Test (BBT) [20, 28, 33, 34], or basic grasp and lift tasks [24, 35–38]. Some of these studies have identified similarities between simulated prosthesis results and prior myoelectric prosthesis user scores [9, 11, 34]. However, task performance scores do not capture important details about participants’ compensatory movements [29].

Other studies have used data from participants using both simulated and actual prosthetic devices, but did not aim to provide a detailed comparison of hand and upper body kinematics between such groups or to compare movement results to unimpaired (normative) limb function. Amsuess et al. included participants using simulated and actual prosthetic devices to compare various device control algorithms [39]. Task scores and durations were measured in this research, but the prosthesis users performed SHAP, whereas the non-disabled participants performed three other assessment tasks. Therefore, comparison between prosthesis users’ and non-disabled participants’ task performance was infeasible. Brown et al. used both such participant groups to investigate the effect of sensory feedback, and identified similar grasping slip measures between the two groups [37]. Sobuh et al. included both types of participants to study visuomotor behaviour and discovered that the two groups had similar gaze fixations, task durations, and SHAP scores [31]. Collectively, these studies have identified some parallels between simulated and actual prosthesis use.

Given that the terminal device of a simulated prosthesis is positioned at an offset (not in the expected location of the hand), non-disabled participants often need to use additional, unusual shoulder and trunk movements to complete tasks [8, 20, 33, 40, 41]. Recognizing such requirements, several studies have recommended that future work include testing with actual myoelectric prosthesis users [28, 32, 36, 42–45], implying that the use of simulated prostheses as proxies for actual myoelectric devices is not yet fully validated. Specifically, it has not been addressed whether or not non-disabled individuals fitted with simulated myoelectric prostheses mimic the compensatory movements of those who use actual myoelectric devices.

To assess the compensatory movements of non-disabled individuals wearing a simulated device, the Gaze and Movement Assessment (GaMA) can be employed. GaMA is a validated functional assessment that uses motion capture and eye tracking to facilitate the recording of end effector and angular kinematic data, along with gaze data [46–49]. Already, a normative dataset with kinematic measures from 20 non-disabled participants (with typical arm function) performing two standard object transfer tasks exists [46, 47]. In addition, compensatory movements of actual myoelectric prosthesis users have been quantified by comparing their results (using the same two object transfer tasks) to those of the normative dataset [2, 50]. A similar comparative approach could be taken with non-disabled individuals wearing a simulated prosthesis to identify their compensations in relation to typical arm function.

Therefore, the goal of this study was to compare compensatory movements exhibited by individuals wearing a simulated prosthesis to those of three myoelectric prosthesis users with transradial amputation. GaMA was used for kinematic data collection in both the simulated and myoelectric prosthesis participant groups, from which performance metrics, hand movements, and upper body angular kinematics were derived. Then, for each participant group, comparisons to the normative baseline’s performance metrics, hand movements, and upper body angular kinematics were used to identify how the movement strategies differed from expected typical arm function. Ultimately, this study aimed to validate the research practice of using non-disabled participants wearing a simulated prosthesis as a proxy for actual prosthesis users, by examining the degree to which their movements differed, and in which direction, from expected normative performance for the same functional tasks.

## Methods

### Simulated prosthesis design

In this study, the simulated sensory motor prosthesis developed by Kuus et al. [12] was used. It was designed to be worn by non-disabled individuals to simulate the function of a myoelectric prosthesis worn by an individual with a right-arm transradial amputation. The simulated prosthesis consists of: a rigid brace to immobilize the wearer’s wrist and hand; two electrodes (electrode model: 13E200 = 60; Otto Bock Healthcare Products; Duderstadt, Germany) to read electromyography signals from the user’s forearm muscles; and a myoelectric hand (MyoHand VariPlus Speed model: 8e38 = 9-R7 1⁄4; Otto Bock Healthcare Products) mounted underneath the brace in the approximate location of the participant’s real hand, with a slight radial offset to provide a participant with a sight line to the terminal device. The simulated prosthesis wearer controls the device by activating their wrist extensor muscles to open the hand, and the wrist flexor muscles to close the hand. Although this simulated sensory-motor prosthesis was originally designed to investigate the impact of sensory feedback [12], it was used in this study to solely examine motor control.

### Participants

A group of 12 non-disabled individuals were recruited to perform a functional task while wearing the simulated prosthesis (hereafter referred to as ‘SP participants’). These participants had no upper-body pathology or history of neurological or musculoskeletal injuries within the past two years. All SP participants were right-handed, 11 were male, with an average age of 23.8 ± 3.4 years (mean ± standard deviation) and an average height of 176.2 ± 6.2 cm.

Three individuals with transradial amputations were recruited to perform the same functional task while wearing their usual, custom-fitted myoelectric prosthesis (hereafter referred to as ‘MP participants’—‘P1′, ‘P2′, and ‘P3′). To determine the pre-task skill level of each participant, the Assessment of Capacity for Myoelectric Control (ACMC) [17] was administered by a trained occupational therapist. ACMC was chosen since it is a well-validated assessment of skill level for myoelectric prosthesis users. The attributes and assessment scores of the MP participants are shown in Table 1.

**Table 1:** Attributes of the MP participants

Attributes	MP participants		
	P1	P2	P3
Age (years)	41	52	37
Gender	F	M	M
Height (cm)	170	184	167
Hand dominance before amputation	Right	Left	N/A (congenital)
Amputation side	Right	Left	Left
Time between amputation and data collection	11 months	18 years	37 years
Hours of prosthesis use per day	10	13	10
Prosthetic Hand	i-Limb	i-Limb	MyoHand VariPlus Speed
ACMC score	44.6	59.1	62.0

The study was approved by the University of Alberta Health Research Ethics Board (Pro00054011), the Department of the Navy Human Research Protection Program (DON-HRPP), and the SSC-Pacific Human Research Protection Office (SSCPAC HRPO). Each participant provided written informed consent.

### Functional task

**Fig. 1:**
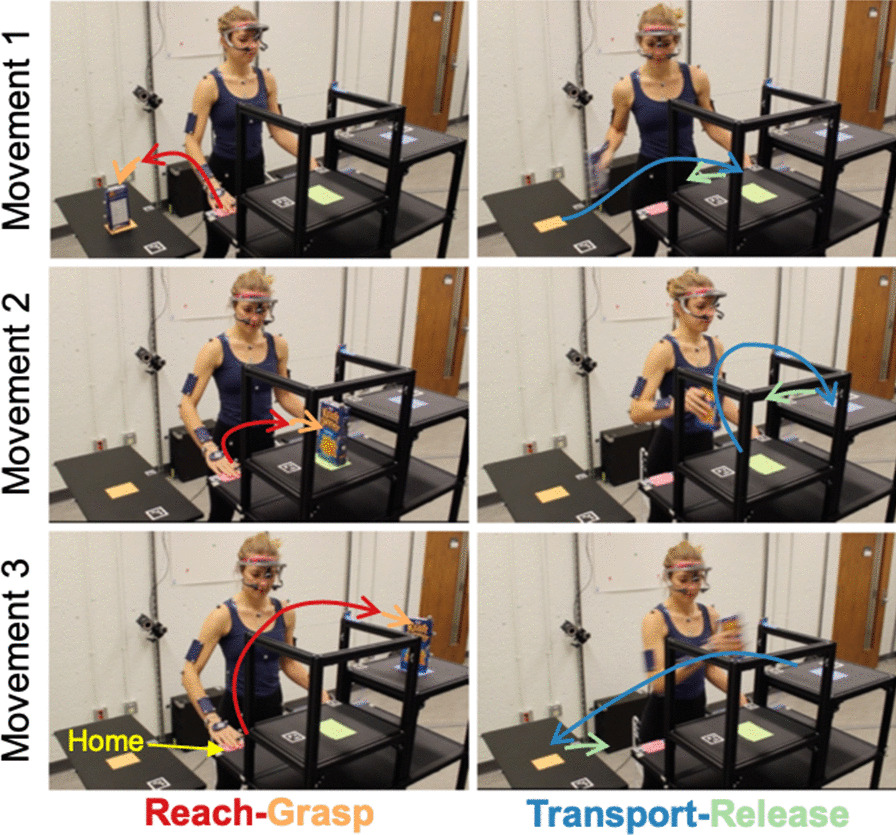
Pasta Box Task. Sequence of the Pasta Box Task movements (Movements 1, 2, and 3) with the ‘home’ position labelled. Reach-Grasp and Transport-Release movement segments are colour-coded and illustrated with arrows to show direction. Although this figure shows a normative participant wearing an eye tracking device, eye gaze behaviour data were not analyzed in this study. Reproduced from Valevicius et al. [46] with permission

The Pasta Box Task, developed by Valevicius et al. [46], validated by Williams et al. [51], and used in prior prosthesis user studies [2, 50], mimics the actions of reaching for a kitchen item and moving it to shelves of different heights – thereby including common prosthesis assessment requirements. In this task, the participant is required to perform three movements: *Movement 1* – moving a pasta box from a lower side table immediately to their right (height: 30 inches) to a shelf in front of them (height: 43 inches); *Movement 2* – moving the pasta box to a second shelf at a higher height across the body (height: 48 inches); and *Movement 3* – moving the pasta box back to the starting position on the side table. The participant is required to start each movement with their hand at a ‘home’ position, and then return their hand to this position at the completion of the task. Each movement, as well as the location of ‘home’, are depicted in Fig. 1. Following data collection, each movement can be divided into the phases of ‘Reach’, ‘Grasp’, ‘Transport’, and ‘Release’, so that discrete characteristics of hand movement can be examined [46]. For our analyses, these phases can be grouped into ‘Reach-Grasp’ and ‘Transport-Release’ movement segments. Note that Fig. 1 shows the Pasta Box Task setup arranged for SP participants (who used the right-side simulated device) and the MP participant with a right-side prosthesis; however, the setup was mirrored for the two MP participants with a left-side prosthesis.

### Prosthetic device training

Each of the SP participants took part in a two-hour device usage training session. During the session, these participants donned the device, were taught how to control the myoelectric hand using their muscle activity, and were given an opportunity to practice functional tasks (including the Pasta Box Task). As the participants carried out these tasks, they were provided with verbal instructions regarding how to improve the control of their device. The participants were allowed to take breaks throughout their training session, as required.

Given that the MP participants were to perform the functional testing with their usual prostheses, they did not require a device usage training session, but were allowed to practice the Pasta Box Task until they felt comfortable executing it.

### SP participant experimental setup

**Fig. 2:**
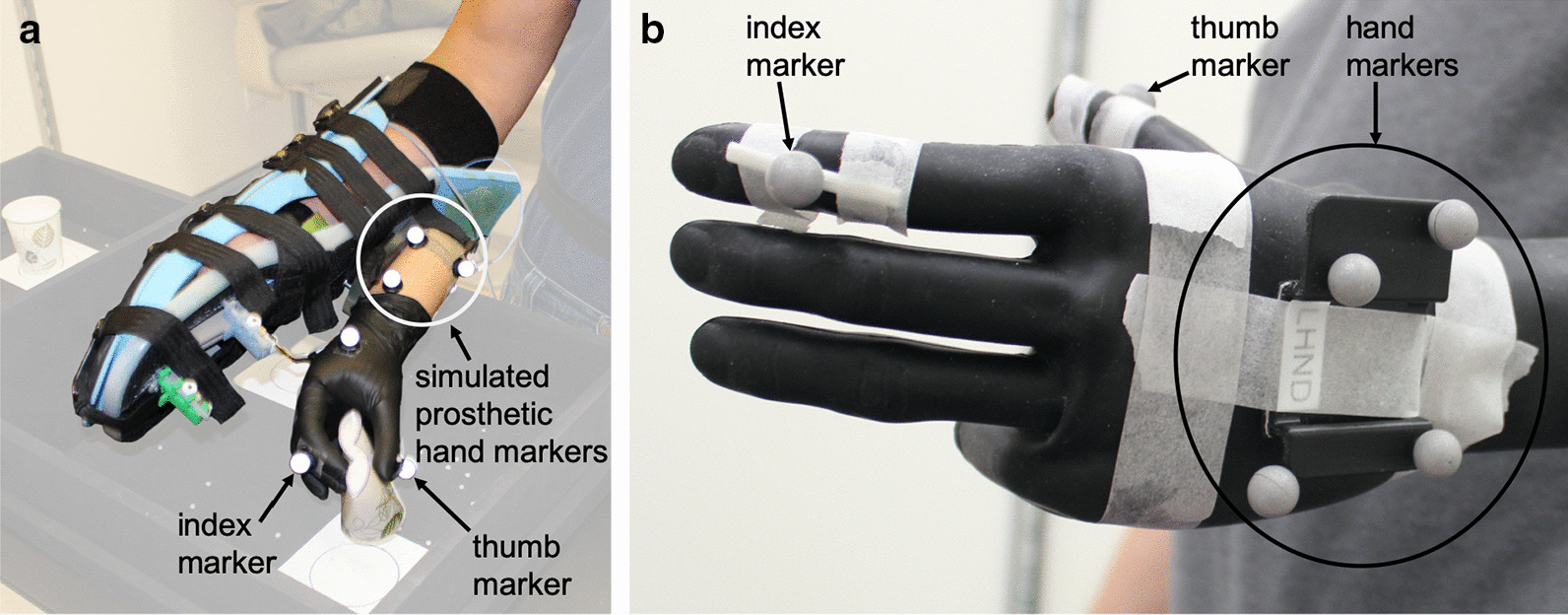
Motion capture marker placement. Placement on the simulated prosthesis (**a**), and a myoelectric prosthesis (**b**). The unlabelled marker on the simulated prosthesis in panel (**a**) was not used for analysis in this study

A 12-camera Vicon Bonita motion capture system (Vicon Motion Systems Ltd, Oxford, UK) was used to capture the three-dimensional trajectories of motion capture markers affixed to the SP participants at a sampling frequency of 120 Hz. Three individual motion capture markers were affixed to a rigid surface of the simulated prosthesis, along with additional markers on the index finger (middle phalange) and thumb (distal phalange), as shown in Fig. 2a. In accordance with Boser et al.’s *Clusters Only* model, rigid plates, each holding four markers, were placed on the participants’ upper arm, trunk, and pelvis [52]. Additional individual markers were placed on the pasta box, shelving unit, and side table, as outlined in the supplementary materials of Valevicius et al. [46].

### MP participant experimental setup

An 8-camera Optitrack Flex 13 motion capture system (Natural Point, OR, USA) was used to capture the three-dimensional trajectories of motion capture markers affixed to the MP participants at a sampling frequency of 120 Hz. While these data were collected at a later date and at a different site than the SP participant data, it should be noted that the reproducibility of the protocol and kinematic results across different motion capture technologies have been previously confirmed [51]. A rigid plate holding four motion capture markers was affixed to the back of each MP participant’s myoelectric hand, along with individual markers on their index finger and thumb as shown in Fig. 2b. As with the SP participants, rigid plates holding four markers were placed on the upper arm, trunk, and pelvis. Additional individual markers were placed on the pasta box, shelving unit, and side table, as outlined in the supplementary materials of Valevicius et al. [46].

### Experimental data acquisition and processing

Before each participant performed the functional task, a motion capture calibration using a modified anatomical pose was performed, as outlined by Boser et al. [52]. In this modified anatomical pose, the participant’s shoulder was at zero degrees of abduction, and the axes passing through the epicondyles and radial and ulnar styloids were aligned with the frontal plane. Then, trial data were collected as follows.

SP participants: Each of the twelve SP participants performed a total of 5 task trials. If they made an error during a trial, the error was flagged, and that trial’s data were discarded. Task errors included dropping the box, an incorrect grasp, an incorrect placement of the box, a missed box drop-off, an incorrect task sequence, hitting the task cart frame, a movement hesitation, or an undesired movement (such as a sneeze). All data from one SP participant were discarded due to poor data quality. Data from a total of 46 trials (from eleven participants) were used in this study.

MP participants: The goal was to obtain up to 20 completed trials for each MP participant. However, if multiple error trials were noted in sequence, or fatigue or frustration were noted due to inability to complete the task, the trial collection was stopped. This resulted in a different number of completed trials for each MP participant: P1 performed 8 trials, with 4 error-free; P2 performed 10 trials, with 4 error-free; and P3 performed 20 trials, with 19 error-free. All error-free trials were used in this study (total of 27 trials across MP participants).

The motion capture data were filtered and segmented into Reach, Grasp, Transport, and Release phases, as outlined by Valevicius et al. [46]. The duration of each phase and relative duration of each phase were calculated. For the simulated and myoelectric prosthetic hands, a rigid body was created using the respective hand markers (the three markers on the side of the simulated prosthesis, shown in Fig. 2a, or the four markers on the back of the myoelectric prostheses, shown in Fig. 2b). Then, a virtual rectangular prism was created to represent the hand object, relative to the rigid body but with an offset so its position would be representative of the simulated or myoelectric hand’s position. Hand movement measures were calculated using the centre of the virtual hand object’s three-dimensional position and its velocity. Time-normalized plots of hand velocity were generated, by normalizing the task length for each trial to 100%, as described by Valevicius et al. [46]. Hand movement measures of peak hand velocity, percent-to-peak hand velocity (percent of time elapsed in a given movement segment before the peak hand velocity occurred), hand distance travelled, hand trajectory variability (maximum of three-dimensional standard deviation at each point in time), and number of movement units (number of velocity peaks) were calculated for each Reach-Grasp and Transport-Release movement segment, as per Valevicius et al. [46]. Grip aperture was measured as the distance between the index and thumb markers, and time-normalized plots of grip aperture were generated, in the same manner as the time-normalized hand velocity plots, as described by Valevicius et al. [46]. Angular kinematics of the shoulder and trunk degrees of freedom (DOFs) were calculated, as outlined by Boser et al. [52]. For each task movement (Movements 1, 2, and 3), ranges of motion (ROMs) were calculated for shoulder and trunk DOFs.

### Data analysis

The three MP participants were represented as individual case studies and mean values across trials for each measure were calculated separately for P1, P2, and P3. For the population of SP participants, an overall mean value was calculated for each measure by averaging across trials and participants. The resulting mean SP participant measures and the individual mean MP participant measures were then compared to those from a normative baseline dataset. This dataset originated from a study conducted by Valevicius et al. and included corresponding mean measures obtained from 20 non-disabled participants who completed the same Pasta Box Task [46, 47]. The non-disabled normative baseline group included: 9 females and 11 males, 18 right-handed and 2 left-handed, 25.8 ± 7.2 years old (mean ± standard deviation), 173.8 ± 8.3 cm tall, each of whom completed 20 task trials. Comparisons between the mean measures from this study and those from the normative baseline dataset facilitated the identification of any compensatory movements introduced through prosthetic device use (be it simulated or actual). That is, a difference in such measures signaled a deviation from normative movements.

All measures from both the SP and normative participant datasets followed a normal distribution, as determined through the use of the Kolmogorov–Smirnov test. To investigate differences between these two groups, a series of mixed analyses of variance (ANOVAs) and pairwise comparisons were conducted for each measure and task. Mixed ANOVA group effects or interactions involving group were followed up with either an additional mixed ANOVA or pairwise comparisons between groups if the Greenhouse–Geisser corrected *p* value was less than 0.05. Pairwise comparisons were considered to be significant if the Bonferroni corrected *p* value was less than 0.05.

All measures from the MP participants were individually compared to the normative baseline as individual case studies. Based on the commonly used convention of defining the normative reference range as two standard deviations above or below the mean [53], the individual P1, P2, and P3 means for each measure were assessed as different from that of the normative baseline if they fell outside of two standard deviations (between-participant) of the corresponding normative mean.

## Results

### Phase duration

**Fig. 3:**
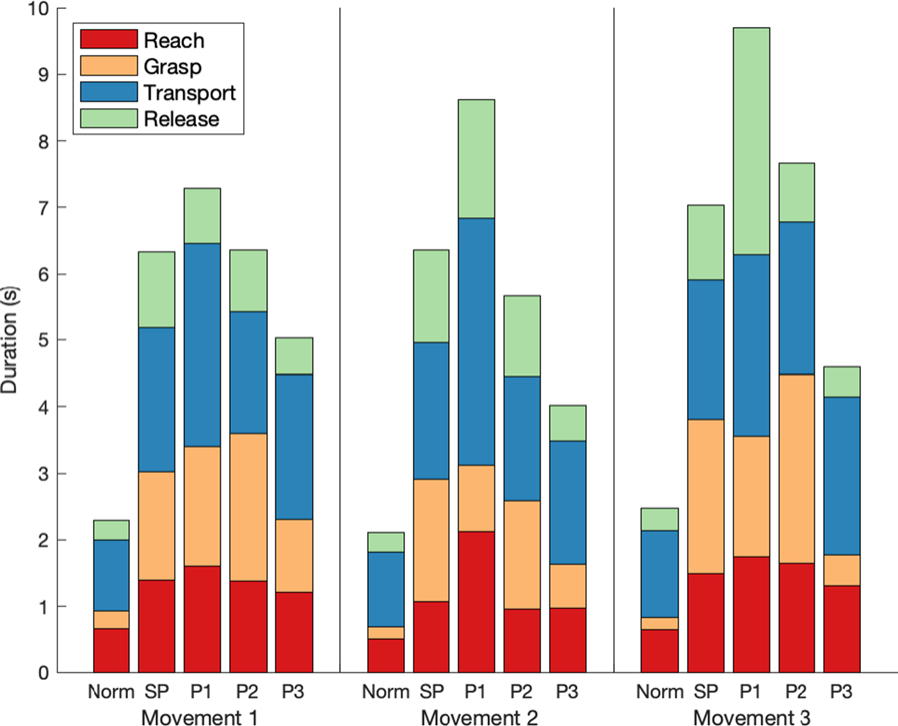
Phase durations. Average Pasta Box Task durations of normative participants (‘Norm’), SP participants, and the three MP participants (P1, P2, P3). These durations are presented for each movement of the task and are divided into Reach, Grasp, Transport, and Release phases, color coded as per legend

The SP participants had an average overall task duration of 24.5 ± 2.8 s, which was significantly longer than the normative duration of 8.8 ± 1.2 s [46] (*p* < 0.01). The three MP participants (P1, P2, and P3) had average overall task durations of 32.7 ± 2.8, 25.5 ± 4.1, and 18.8 ± 0.7 s, respectively, which were all more than two standard deviations larger than the normative mean. As shown in Fig. 3, the SP participants had similar phase durations to P2. P1 typically took more time to complete each phase versus the SP participants, whereas P3 took less time (although they still took more time than normative participants). As shown in Table 2, the SP participants had significantly longer durations than the normative baseline for all phases. The MP participants also had durations that were more than two standard deviations longer than the normative mean for all phases.

**Table 2:** Duration and hand movement measures

		**Duration (sec)**					**Relative duration (%)**				
		ND	SP	P1	P2	P3	ND	SP	P1	P2	P3
M 1	R	0.66 ± 0.08	↑ 1.39 ± 0.28**	↑ 1.61 ± 0.21	↑ 1.39 ± 0.09	↑ 1.21 ± 0.11	29.0 ± 2.0	↓ 22.6 ± 4.4**	↓ 22.0 ± 1.9	↓ 22.0 ± 3.3	↓ 24.3 ± 2.5
	G	0.27 ± 0.08	↑ 1.63 ± 0.53**	↑ 1.80 ± 0.21	↑ 2.21 ± 0.47	↑ 1.10 ± 0.37	11.5 ± 2.5	↑ 25.5 ± 5.9**	↑ 24.6 ± 1.7	↑ 34.4 ± 4.2	↑ 21.6 ± 6.0
	T	1.08 ± 0.12	↑ 2.15 ± 0.50**	↑ 3.06 ± 0.62	↑ 1.83 ± 0.21	↑ 2.17 ± 0.19	47.1 ± 2.2	↓ 34.0 ± 4.0**	↓ 42.3 ± 9.8	↓ 28.7 ± 1.5	43.4 ± 5.0
	RL	0.28 ± 0.07	↑ 1.16 ± 0.43**	↑ 0.82 ± 0.55	↑ 0.94 ± 0.16	↑ 0.54 ± 0.15	12.4 ± 2.3	↑ 17.9 ± 3.3**	11.1 ± 6.8	14.9 ± 2.6	10.8 ± 3.1
M 2	R	0.52 ± 0.06	↑ 1.07 ± 0.17**	↑ 2.12 ± 0.70	↑ 0.96 ± 0.08	↑ 0.97 ± 0.09	24.4 ± 2.0	↓ 17.2 ± 2.6**	↓ 25.0 ± 7.4	↓ 17.0 ± 1.1	24.2 ± 2.8
	G	0.18 ± 0.05	↑ 1.84 ± 0.43**	↑ 1.00 ± 0.40	↑ 1.63 ± 0.22	↑ 0.67 ± 0.15	8.3 ± 1.7	↑ 28.6 ± 5.7**	11.5 ± 2.8	↑ 29.1 ± 5.2	↑ 16.4 ± 2.7
	T	1.12 ± 0.13	↑ 2.05 ± 0.45**	↑ 3.71 ± 1.45	↑ 1.86 ± 0.23	↑ 1.84 ± 0.16	53.0 ± 2.9	↓ 32.8 ± 4.9**	↓ 43.0 ± 11.2	↓ 32.8 ± 2.0	↓ 45.8 ± 2.6
	RL	0.30 ± 0.08	↑ 1.40 ± 0.61**	↑ 1.78 ± 0.94	↑ 1.21 ± 0.50	↑ 0.55 ± 0.08	14.2 ± 2.7	↑ 21.4 ± 6.6**	↑ 20.6 ± 10.2	↑ 21.1 ± 7.7	13.5 ± 1.4
M 3	R	0.65 ± 0.10	↑ 1.49 ± 0.35**	↑ 1.75 ± 0.52	↑ 1.64 ± 0.57	↑ 1.31 ± 0.10	26.2 ± 1.8	↓ 21.3 ± 3.8*	↓ 17.9 ± 4.8	↓ 22.0 ± 3.5	28.5 ± 1.5
	G	0.19 ± 0.06	↑ 2.32 ± 0.61**	↑ 1.81 ± 0.28	↑ 2.84 ± 2.45	↑ 0.46 ± 0.10	7.4 ± 1.8	↑ 32.5 ± 5.3**	↑ 18.6 ± 2.7	↑ 32.9 ± 13.7	10.1 ± 1.7
	T	1.31 ± 0.16	↑ 2.10 ± 0.38**	↑ 2.74 ± 0.24	↑ 2.30 ± 0.31	↑ 2.36 ± 0.31	52.9 ± 2.1	↓ 30.5 ± 4.5**	↓ 28.3 ± 3.1	↓ 32.3 ± 7.5	51.4 ± 6.1
	RL	0.34 ± 0.07	↑ 1.13 ± 0.55**	↑ 3.40 ± 0.18	↑ 0.87 ± 0.28	↑ 0.46 ± 0.28	13.6 ± 2.2	15.7 ± 5.9	↑ 35.2 ± 2.7	12.8 ± 5.9	10.0 ± 5.9
		**Peak hand velocity (mm/s)**					**Percent-to-peak hand velocity (%)**				
		ND	SP	P1	P2	P3	ND	SP	P1	P2	P3
M 1	RG	1164 ± 163	↓ 812 ± 107**	873 ± 117	1038 ± 132	↓ 714 ± 61	41.2 ± 4.5	↓ 25.8 ± 4.6**	↓ 17.7 ± 2.4	↓ 16.3 ± 1.7	↓ 27.3 ± 5.7
	TRL	1447 ± 136	↓ 1057 ± 188**	↓ 682 ± 33	↓ 1053 ± 179	↓ 860 ± 47	29.3 ± 3.1	↓ 22.1 ± 5.5**	28.4 ± 3.8	↓ 22.2 ± 4.4	↑ 37.2 ± 5.1
M 2	RG	1352 ± 191	↓ 927 ± 195**	↓ 663 ± 32	1018 ± 96	↓ 702 ± 62	36.8 ± 4.4	↓ 11.9 ± 2.0**	↓ 14.1 ± 3.8	↓ 9.9 ± 0.7	↓ 17.0 ± 3.1
	TRL	1069 ± 112	↓ 779 ± 172**	↓ 463 ± 64	971 ± 99	↓ 661 ± 49	44.8 ± 8.6	↓ 32.5 ± 8.4**	36.2 ± 10.2	32.8 ± 7.5	48.5 ± 5.4
M 3	RG	1666 ± 261	↓ 1267 ± 277**	↓ 1030 ± 199	1389 ± 125	↓ 1104 ± 98	35.5 ± 4.0	↓ 11.7 ± 2.4**	↓ 16.1 ± 2.1	↓ 11.4 ± 5.7	↓ 18.1 ± 2.0
	TRL	1598 ± 180	↓ 1343 ± 267*	↓ 931 ± 47	1432 ± 160	↓ 1210 ± 87	36.2 ± 3.8	35.4 ± 8.6**	↓ 24.4 ± 1.4	37.8 ± 4.9	↑ 44.1 ± 2.7
		**Hand distance travelled (mm)**					**Hand trajectory variability (mm)**				
		ND	SP	P1	P2	P3	ND	SP	P1	P2	P3
M 1	RG	492 ± 26	↑ 747 ± 58**	↑ 745 ± 51	↑ 957 ± 31	↑ 595 ± 30	19 ± 5	↑ 49 ± 18**	↑ 32	↑ 46	↑ 44
	TRL	935 ± 27	↑ 1003 ± 42**	↑ 1043 ± 10	↑ 1097 ± 28	912 ± 24	22 ± 4	↑ 72 ± 40**	↑ 54	↑ 44	↑ 52
M 2	RG	505 ± 23	↑ 545 ± 31**	↑ 627 ± 138	↑ 615 ± 21	↓ 428 ± 12	15 ± 5	↑ 38 ± 19**	↑ 48	18	16
	TRL	802 ± 61	↑ 957 ± 70**	↑ 969 ± 150	↑ 977 ± 61	832 ± 30	20 ± 4	↑ 58 ± 48**	↑ 83	↑ 121	15
M 3	RG	746 ± 24	↑ 953 ± 77**	↑ 1075 ± 12	↑ 1110 ± 252	748 ± 25	19 ± 4	↑ 68 ± 29**	↑ 40	↑ 153	23
	TRL	1186 ± 31	↑ 1407 ± 63**	↑ 1723 ± 105	↑ 1462 ± 22	↑ 1324 ± 23	35 ± 8	↑ 106 ± 55**	↑ 79	↑ 101	↑ 52
		**Number of movement units**									
		ND	SP	P1	P2	P3					
M 1	RG	1.3 ± 0.3	↑ 9.8 ± 3.4**	↑ 8.8 ± 2.4	↑ 10.3 ± 3.9	↑ 4.7 ± 1.6					
	TRL	1.2 ± 0.2	↑ 8.4 ± 3.1**	↑ 6.0 ± 1.4	↑ 5.3 ± 1.0	↑ 3.8 ± 1.3					
M 2	RG	1.0 ± 0.1	↑ 11.0 ± 3.7**	↑ 9.0 ± 1.8	↑ 6.8 ± 1.9	↑ 3.3 ± 1.0					
	TRL	2.3 ± 0.4	↑ 11.1 ± 3.6**	↑ 11.3 ± 6.1	↑ 7.5 ± 3.1	↑ 4.3 ± 1.2					
M 3	RG	1.1 ± 0.1	↑ 15.7 ± 4.9**	↑ 9.3 ± 1.5	↑ 15.5 ± 12.2	↑ 3.4 ± 1.2					
	TRL	1.7 ± 0.4	↑ 8.2 ± 3.6**	↑ 13.0 ± 2.4	↑ 5.8 ± 1.3	↑ 4.1 ± 0.7					

Non-disabled (ND) baseline and SP group duration and hand movement means and across-participant standard deviations, and MP participant (P1, P2, P3) means and standard deviations for each movement (M) and phase (Reach: R, Grasp: G, Transport: T, Release: RL) or movement segment (RG or TRL). For SP values, pairwise comparison results are indicated with asterisks (* for *p* < 0.05, ** for *p* < 0.005). Arrows indicate that a given SP mean was significantly different from the normative mean, or that an MP mean was outside of two standard deviations of the non-disabled mean (↑ indicating higher and ↓ indicating smaller)

As shown in Table 2, most deviations from normative values trended in the same direction for the SP and MP participants. The SP participants had significantly longer relative phase durations than the normative baseline for all Grasp and most Release phases, and, consequently, significantly shorter relative phase durations for all Reach and Transport phases. P1 and P2 exhibited this trend throughout most of the task, although P3 only exhibited this trend in four phases, with the majority of their relative phase durations within two standard deviations of the normative baseline).

### Hand velocity

**Fig. 4:**
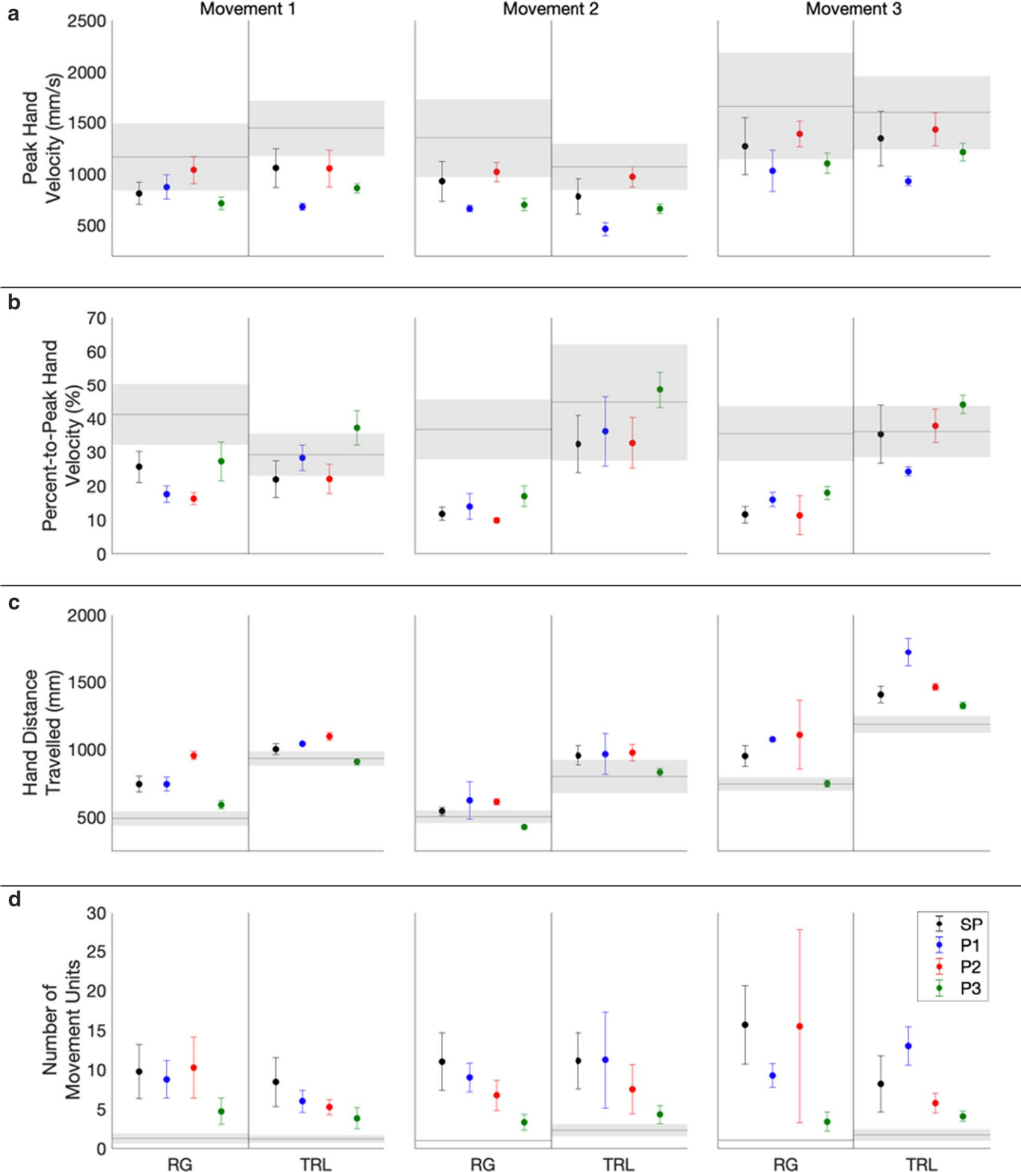
Hand movement measures. Peak hand velocity (**a**), percent-to-peak hand velocity (**b**), hand distance travelled (**c**), and number of movement units (**d**) of the SP participants (black) and the MP participants (P1: blue, P2: red, P3: green), for each task movement and movement segment (*RG* Reach-Grasp, *TRL* Transport-Release). Dots indicate the average value for each movement segment, and each error bar represents ± 1 standard deviation (between-participant standard deviation is presented for SP participants). The ranges of motion of a normative baseline [47] are presented with grey lines representing the average and with shading representing ± 2 between-participant standard deviations

Table 2 identifies that the SP participants exhibited significantly smaller hand velocity peaks than the normative baseline throughout the task, since they performed the task slower. P3 exhibited this trend throughout the task, as did P1 during most movement segments, although P2 generally had peak hand velocity means that were closer to those of the normative baseline. The deviation of the SP and MP participants’ peak hand velocities from normative means is also shown in Fig. 4a, with all such values below the normative means.

The SP participants exhibited significantly earlier hand velocity peaks than the normative baseline in all Reach-Grasp movement segments, and the MP participants all exhibited this trend throughout the task (Fig. 4b, Table 2). For Transport-Release movement segments, most SP and MP data points for percent-to-peak hand velocity were within or close to two standard deviations of the normative means (Fig. 4b, Table 2).

### Hand trajectory

The SP participants had significantly greater hand distances travelled than the normative baseline throughout the task. P1 and P2 also had hand distances travelled that were more than two standard deviations greater than the normative means (Table 2, Fig. 4c). P3, however, only exhibited this trend in two movement segments.

SP participants had significantly greater hand trajectory variability than the normative baseline throughout the task. P1 also exhibited this trend throughout the task, and P2 exhibited this trend for most of the task. However, P3 only followed this trend in half of the movement segments.

Finally, the SP participants had a significantly larger number of movement units than the normative baseline in all movement segments. All three MP participants also had more than two standard deviations more movement units than the normative means throughout the task (Fig. 4d).

### Grip aperture

**Fig. 5:**
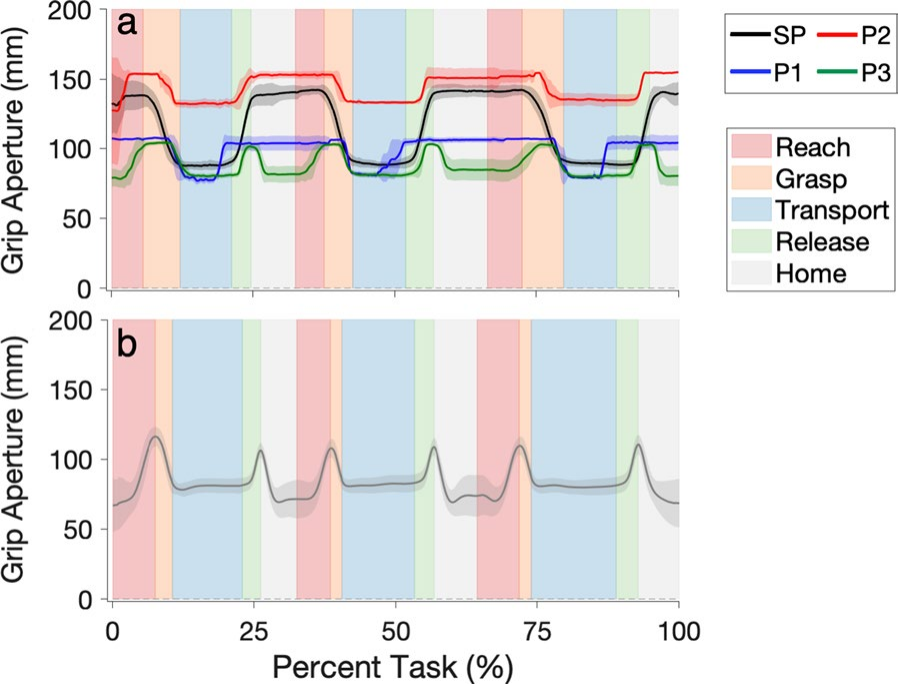
Grip aperture profiles. Profiles of the SP participants (black) and of the MP participants (P1: blue, P2: red, P3: green) (**a**) and of the normative baseline [46] (grey, **b**) over the course of the Pasta Box Task (all 3 movements). The solid lines represent averages and the shading represents ± 1 standard deviation (between-participant standard deviation is presented for SP participants). The average (all SP and MP participants) relative durations of each phase (Reach, Grasp, Transport, Release, Home) can be inferred from the width of the corresponding colored bars. Grip aperture profiles were time normalized by phase and resampled using these average relative phase durations. Normative grip aperture plots are shown in a separate panel due to the differences between normative relative phase durations and those of the SP and MP participants

The grip aperture profiles of the SP and MP participants (Fig. 5a) were all visually different from those of normative participants (Fig. 5b), demonstrating a plateau during Reach phases. P2′s grip aperture profile was most comparable to that of the SP participants, with the exception of the grip aperture magnitudes during Transport phases. This finding is explained by the observation that P2 could only successfully complete the task by grasping the 7-inch × 3.5-inch × 1.5-inch pasta box by its 3.5-inch side to perform the task, rather than its 1.5-inch side, in order to complete the task successfully. P1′s grip aperture profile was also made up of plateaus at hand open or hand closed, although that participant exhibited early hand opening before the end of the Transport phase; P1 placed the pasta box *close to* the desired targets and then pushed the box to these locations. Finally, P3′s grip aperture profile also contained plateaus, although they closed the hand while moving it back to the home location, similar to the normative individuals.

### Angular kinematics

**Fig. 6:**
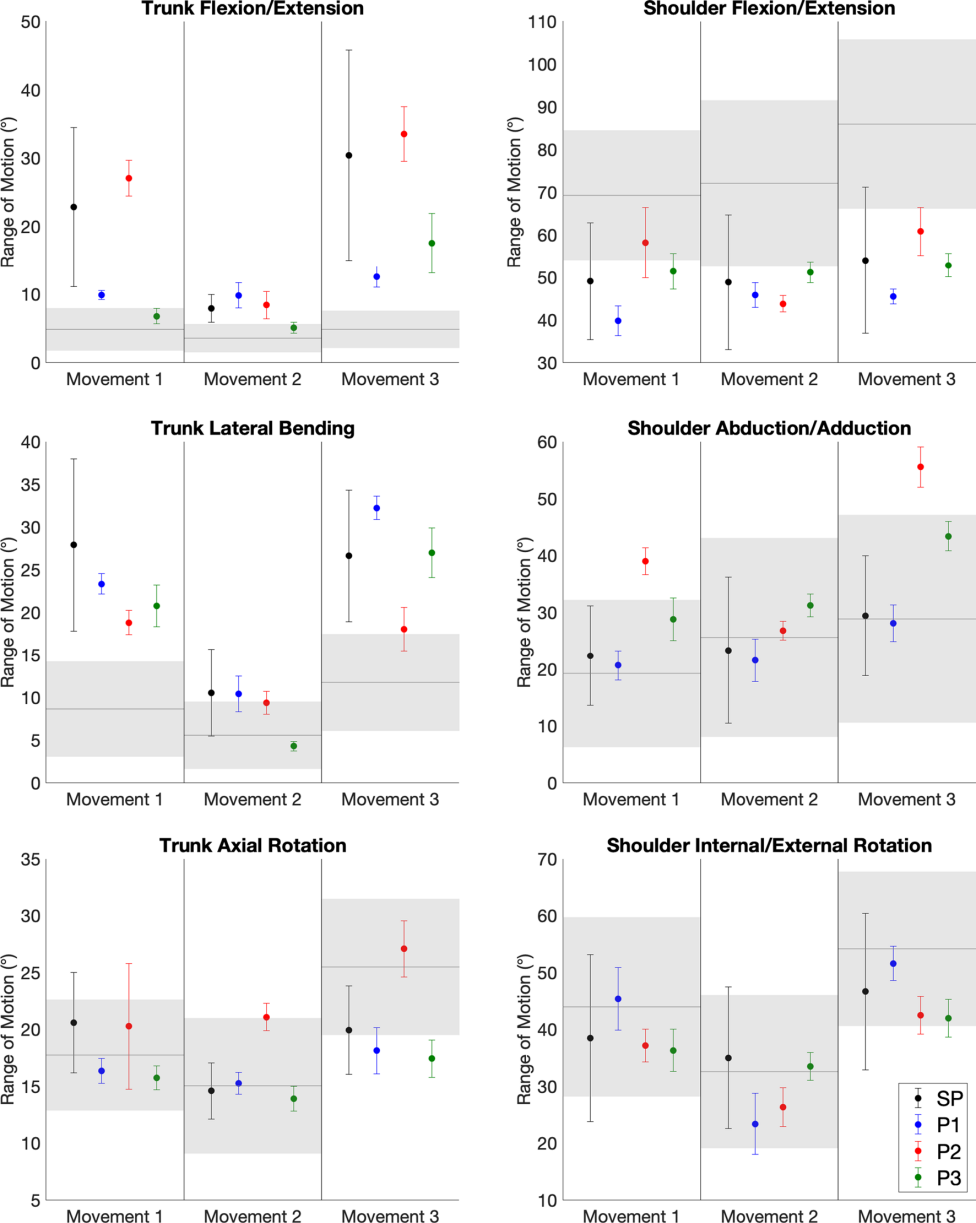
Angular kinematic ranges of motion. Ranges of motion of the SP participants (black) and the MP participants (P1: blue, P2: red, P3: green), for each degree of freedom and each task movement. Dots indicate the average range of motion for each movement, and each error bar represents ± 1 standard deviation (between-participant standard deviation is presented for SP participants). The ranges of motion of a normative baseline [47] are presented with grey lines representing the average and with shading representing ± 2 between-participant standard deviation

Figure 6 illustrates the ROM values for the SP participant group and three MP participants, for trunk and shoulder DOFs, as well as the normative baseline. As shown in Table 3, the SP participants exhibited significantly greater ROMs in trunk flexion/extension and lateral bending throughout the task when compared to the normative baseline, and significantly smaller ROMs in shoulder flexion/extension throughout the task and trunk axial rotation in movement 3. As shown in Table 3 and in Fig. 6, P1 exhibited these same differences, as their mean ROMs in these DOFs and movements were outside of 2 standard deviations from the corresponding normative means. P3 exhibited these ROM trends in trunk axial rotation and shoulder flexion/extension, as well as for trunk flexion/extension in Movement 3 and in trunk lateral bending in Movements 1 and 3. P2 also exhibited the same ROM trend in trunk flexion/extension, trunk lateral bending in Movements 1 and 3, and in shoulder flexion/extension Movements 2 and 3. However, P2 exhibited greater ROMs in trunk axial rotation in Movement 2 and in shoulder abduction/adduction in Movements 1 and 3. It should also be noted that the SP participants generally exhibited large variability in their ROMs (e.g. in shoulder flexion/extension in all movements, and in trunk flexion/extension in Movements 1 and 3), indicating that the participants employed different compensatory movements from each other.

**Table 3:** Angular kinematic ranges of motion

		Range of motion (degrees)				
		ND	SP	P1	P2	P3
Trunk Flexion/Extension	Movement 1	4.9 ± 1.6	↑ 22.8 ± 11.7**	↑ 9.9 ± 0.7	↑ 27.1 ± 2.6	6.8 ± 1.1
	Movement 2	3.6 ± 1.0	↑ 8.0 ± 2.0**	↑ 9.9 ± 1.8	↑ 8.4 ± 2.0	5.1 ± 0.8
	Movement 3	4.9 ± 1.4	↑ 30.4 ± 15.4**	↑ 12.6 ± 1.6	↑ 33.5 ± 4.0	↑ 17.6 ± 4.4
Trunk Lateral Bending	Movement 1	8.7 ± 2.8	↑ 27.9 ± 10.1**	↑ 23.4 ± 1.2	↑ 18.8 ± 1.4	↑ 20.8 ± 2.4
	Movement 2	5.6 ± 2.0	↑ 10.6 ± 5.1**	↑ 10.4 ± 2.1	9.4 ± 1.3	4.3 ± 0.6
	Movement 3	11.8 ± 2.8	↑ 26.6 ± 7.7**	↑ 32.3 ± 1.4	↑ 18.1 ± 2.6	↑ 27.0 ± 2.9
Trunk Axial Rotation	Movement 1	17.8 ± 2.4	20.6 ± 4.4	16.4 ± 1.1	20.3 ± 5.5	15.8 ± 1.1
	Movement 2	15.1 ± 3.0	14.6 ± 2.5	15.3 ± 1.0	↑ 21.1 ± 1.2	13.9 ± 1.1
	Movement 3	25.5 ± 3.0	↓ 19.9 ± 3.9**	↓ 18.2 ± 2.0	27.1 ± 2.5	↓ 17.5 ± 1.6
Shoulder Flexion/Extension	Movement 1	69.3 ± 7.6	↓ 49.1 ± 13.8**	↓ 39.8 ± 3.5	58.2 ± 8.2	↓ 51.5 ± 4.2
	Movement 2	72.1 ± 9.7	↓ 48.9 ± 15.8**	↓ 45.9 ± 2.9	↓ 43.8 ± 1.9	↓ 51.2 ± 2.4
	Movement 3	86.0 ± 9.9	↓ 54.1 ± 17.2**	↓ 45.7 ± 1.5	↓ 60.8 ± 5.6	↓ 52.9 ± 2.7
Shoulder Abduction/Adduction	Movement 1	19.3 ± 6.5	22.4 ± 8.8	20.7 ± 2.6	↑ 39.1 ± 2.3	28.8 ± 3.8
	Movement 2	25.6 ± 8.8	23.4 ± 12.9	21.6 ± 3.7	26.8 ± 1.6	31.2 ± 2.0
	Movement 3	28.9 ± 9.1	29.4 ± 10.6	28.1 ± 3.3	↑ 55.6 ± 3.5	43.4 ± 2.6
Shoulder Internal/External Rotation	Movement 1	44.0 ± 7.9	38.5 ± 14.7	45.4 ± 5.5	37.2 ± 2.9	36.4 ± 3.7
	Movement 2	32.6 ± 6.7	35.0 ± 12.4	23.4 ± 5.4	26.4 ± 3.4	33.5 ± 2.5
	Movement 3	54.2 ± 6.8	46.7 ± 13.8	51.7 ± 3.0	42.5 ± 3.3	42.0 ± 3.3

Non-disabled (ND) baseline and SP group range of motion means and across-participant standard deviations, and MP participant (P1, P2, P3) means and standard deviations for each movement. Ranges of motion were calculated for the following degrees of freedom: trunk flexion/extension, lateral bending, and axial rotation; shoulder flexion/extension, abduction/adduction, and internal/external rotation. For SP values, pairwise comparison results are indicated with asterisks (** for *p* < 0.005). Arrows indicate that a given SP mean was significantly different from the normative mean, or that an MP mean was outside of two standard deviations of the non-disabled mean (↑ indicating higher and ↓ indicating smaller)

## Discussion

This study determined that, in comparison to non-disabled participants, both simulated prostheses (SP) participants and actual myoelectric prostheses (MP) participants use compensatory movements when performing a standardized object transfer task. These compensations were consistent across the various movement phases and task challenges. Prior work that compared the hand function metrics of individuals wearing a simulated prosthesis to those of non-disabled participants [54] demonstrated that simulated device users performed the Pasta Box Task slower, with prolonged Grasp and Release phases, smaller and earlier hand velocity peaks, larger hand distances travelled, increased hand trajectory variability, and more movement units [54]. This study has extended such findings by identifying that compensatory movements exhibited by SP participants during task execution closely resemble those of MP participants.

It was presumed that MP participants may be more adept at device control during task performance, in comparison to the SP participants in this study who had no prior myoelectric control experience. MP participants’ (P1, P2, P3) device control skill levels were evaluated by considering their ACMC scores and the Pasta Box Task completion time. Although the ACMC scores of P2 and P3 indicated that these participants were both “extremely capable”, the difference between their scores was 2.9 and therefore more than the minimal detectable change with the same rater [16]. Task completion time was additionally used to ascertain device control adeptness, given the commonality of speed as a rating criteria in many prosthesis functional outcome measures [55]. From these assessments, P1 was considered to be the “least-skilled”, P2 was “mid-skilled”, and P3 was the “most-skilled”. These particular MP participant skill levels are taken into consideration throughout the remainder of this discussion.

Results of this study reveal that both SP and MP participants took longer to perform the task than non-disabled participants, with the SP phase durations most closely resembling those of the mid-skilled MP participant. This is in keeping with Sobuh et al. observation that individuals wearing simulated prostheses have functional task performance durations that are similar to the average durations of myoelectric prosthesis users [31]. The relative phase durations of the SP participants and least- and mid-skilled MP participants indicated they specifically took longer to grasp and release objects, with less relative time spent reaching and transporting the object. Given that skill level is associated with movement time duration [13], Grasp and Release phases were presumably prolonged because object manipulation (grasping and releasing) is more difficult to master than object transfer. The MP participant that was rated the most skilled by the ACMC expectedly demonstrated more assured object manipulation with less relative prolongation of Grasp and Release (only 2 Grasp phases prolonged).

The SP participants and least- and mid-skilled MP participants had larger hand distances travelled than the non-disabled individuals. The most-skilled MP user, however, had values that were closer to those of the non-disabled participants, which may indicate use of a more efficient Reach and Transport path. The SP participants and least- and mid-skilled MP participants also had larger hand trajectory variability than the normative baseline, whereas the most-skilled MP user only exhibited this trend in half of the movement segments, which may have been indicative of confidence when performing the task. However, all of the SP and MP participants used a greater number of movement units than the non-disabled baseline, indicating a common experience of prosthetic device movement challenges. Additionally, all SP and MP participants had earlier Reach-Grasp hand velocity peaks in comparison to non-disabled participants, indicating a common conservative control strategy [56] and perceived difficulty in grasping an object.

The SP participants had comparable grip aperture profiles to the least- and mid-skilled MP participants, all of which showed a series of plateaus. Additionally, all of these participants displayed an uncoupling of Reach and Grasp, consistent with observations reported by other studies of myoelectric prosthesis use [57]. The grip aperture profile of the most-skilled MP participant was more similar to that of non-disabled participants [58]. This most-skilled participant closed their hand while moving it back to home (rather than keeping it open) and did not exhibit an uncoupling of Reach and Grasp. Despite using this strategy, this participant still exhibited small plateaus when their hand was fully open, which is in keeping with Bouwsema et al.’s observation that myoelectric prosthesis users with higher skill levels exhibit shorter hand open plateaus [13].

The trunk and shoulder kinematic results reveal that SP and MP participants exhibited similar body movement compensations, with larger trunk flexion/extension and lateral bending movements in comparison to non-disabled individuals. These findings are consistent with prior studies of myoelectric prosthesis users performing other functional object manipulation tasks [3, 26, 59]. The SP and MP participants also exhibited smaller shoulder flexion/extension ROMs than non-disabled individuals, in keeping with previous observations of myoelectric [26] and other upper limb prostheses users [2]. Carey et al. noted that the constraint due to a wrist-immobilizing brace while using the intact hand did not produce the same magnitudes of compensatory movements as those introduced by myoelectric prosthesis use [26]. This finding suggests that wrist immobilization alone does not adequately simulate myoelectric prosthesis use, but rather, that myoelectric hand grasp function also affects the compensatory movements observed. Finally, the SP participants exhibited large variability in their ROMs, which is in keeping with Major et al.’s observation that myoelectric prosthesis users tend to exhibit varied kinematic movement strategies between each other [59].

This study was not without limitations. The three MP users recruited for this study may not have been a true representation of the population, although they did present a range of skill levels. Group statistical analyses could not be performed for the MP participants, so additional MP user data could further support the inferences presented, notwithstanding the large heterogeneity of prosthesis users, which may dilute group comparisons. Another influence on the study was the amount of training that the SP participants received, since presumably more practice would result in more efficient movement strategies [60], and may have shifted certain results closer to those of the more skilled prosthesis user. The type of training provided might also explain the large between-participant standard deviation for the ROMs of the SP participants. As the training session involved primarily instruction regarding prosthetic hand grasp control (rather than training on movement strategies), SP participants adopted various trunk and shoulder movements to accomplish the task. The impact of additional training on kinematic strategies would be an important area of future study, as well as determining the optimal amount of practice needed for SP participants to accurately represent the varied skill levels exhibited by prosthesis users. Finally, since only one simulated prosthesis design was used in this study for the execution of one complex functional task, the findings cannot be directly applied to other simulated prostheses with substantially different device designs (e.g., if placement of the terminal device is distal to the arm (31), rather on the palmar side).

## Conclusions

Overall, this study suggests that participants using a simulated prosthesis reach for and transport objects using comparable compensatory movements to those of a low- to moderately-skilled transradial myoelectric user, with respect to performance metrics, hand movements, and upper body angular kinematics. The influence of training and task practice on simulated prosthesis performance requires further investigation, the results of which could create additional profiles of more highly skilled myoelectric prosthesis user counterparts. Broadly, this study provides reassurance for kinematic research that employs simulated devices to study transradial myoelectric prostheses operation. Furthermore, it presents recommendations towards further assessments of the validity of this research practice.

## Data Availability

The datasets generated during and/or analyzed during the current study are available from the corresponding author on reasonable request.

## Abbreviations

ACMC: Assessment of Capacity for Myoelectric Control
ANOVA: Analysis of variance
DOF: Degree of freedom
MP: Myoelectric prosthesis
ROM: Range of motion
SP: Simulated prosthesis
SHAP: Southampton Hand Assessment Procedure
BBT: Box and Blocks Task
GaMA: Gaze and Movement Assessment
